# Supplementary material for: An Immunconjugate Vaccine Alters Distribution and Reduces the Antinociceptive, Behavioral and Physiological Effects of Fentanyl in Male and Female Rats
Source: Pharmaceutics. 2022 Oct 26;14(11):2290. doi: 10.3390/pharmaceutics14112290 (PMC9694531; doi:10.3390/pharmaceutics14112290)
Supplement: Supplementary file 1 [file pharmaceutics-14-02290-s001.zip › pharmaceutics-1920536-supplementary.pdf]

Experimental Procedures: Sufentanil-BSA conjugate Synthesis

1. General Information

All reactions involving air-sensitive reagents were carried out with magnetic stirring and oven-dried glassware with rubber septa under argon unless otherwise stated. All commercially available chemicals and reagent grade solvents were used directly without further purification, unless otherwise specified. Reactions were monitored by thin-layer chromatography (TLC) on Baker-flex® silica gel plates (IB2-F) using UV-light (254 and 365 nm) detection or visualizing agents (e.g., iodine, ninhydrin or phosphomolybdic acid stain). Flash chromatography was conducted on a silica gel (230-400 mesh) using a Teledyne ISCO CombiFalsh® Rf. NMR spectra were recorded at room temperature using a JEOL ECA-600 instrument (^{1}H NMR at 600 MHz and ^{13}C NMR at 151 MHz) with tetramethyl silane (TMS) as an internal standard. Chemical shifts (δ) are given in parts per million (ppm) with reference to solvent signals [^{1}H-NMR: $CDCl_3$ (7.26 ppm), CD_3OD (3.31 ppm), DMSO-d_6 (2.50 ppm); ^{13}C-NMR: $CDCl_3$ (77.0 ppm), CD_3OD (49.0 ppm), DMSO-d_6 (39.5 ppm)]. Signal patterns are reported as s (singlet), d (doublet), t (triplet), q (quartet), qu (quintet), m (multiplet) and br (broad). Coupling constants (J) are given in Hz. High-resolution mass spectra (HRMS) were obtained by the University of Texas, Austin mass spectral facility using a Qstar Elite-ESI and reported as m/z (relative intensity) for the molecular ion [M].

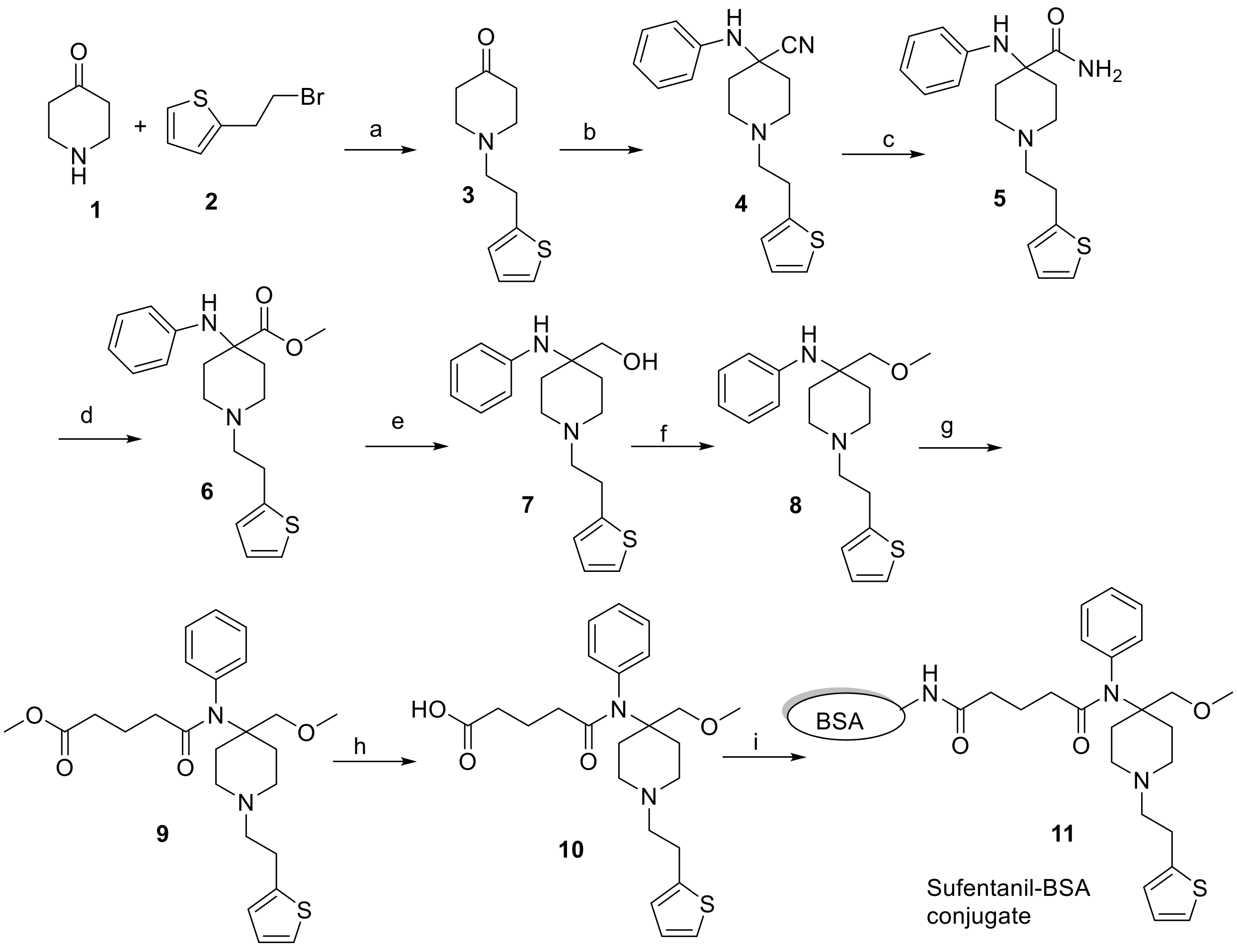

Scheme S1. Reagents and conditions: a) K_2CO_3, CH_3CN, 80 °C, 16 h; b) Aniline, TMSCN, AcOH, 0 °C to rt, 4 h; c) H_2O_2, K_2CO_3, DMSO, rt, 16 h; d) Triflicacid, methanol, DMSO, 0 °C - 80 °C, 2 days; e) $LiAlH_4$, THF, 0 °C to rt, 2 h; f) NaH, THF, 70 °C, 1 h, MeI, THF, 0 °C, 16 h; g) Methyl 4-(chloroformyl)butyrate, py, CH_2Cl_2, 0 °C-70 °C, 16 h; h) $LiOH.H_2O$ (1M), MeOH, rt, 4 h; i) EDC, NHS, DMSO 16 h, BSA in H_2O, 3 h.

1-(2-(Thiophen-2-yl)ethyl)piperidin-4-one (3)

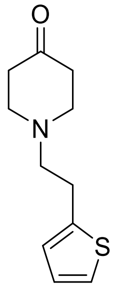

To a solution of **1** (4.2 g, 31.0 mmol), compound **2** (5.3 g, 27.9 mmol) in anhydrous CH_3CN (140 mL) was added K_2CO_3 (12.8 g, 92.9 mmol) at room temperature. The reaction mixture was refluxed in oil bath for 16 h at 80 °C. After completion, the reaction mixture was slowly cooled to room temperature and the reaction mixture filtered through a small Celite pad. The filtrate was washed with water and extracted with ethyl acetate (2 x 50 mL). The combined organic layers were washed with brine solution and dried over hydrous sodium sulfate, filtered and concentrated. The crude compound was purified by combiflash chromatography (0−15% ethyl acetate in hexane) to yield **3** (4.0 g, 61% yield) as a colorless liquid. ^{1}H NMR (600 MHz, $CDCl_3$): δ 7.15 (d, *J* = 5.0 Hz, 1H), 6.94 (t, *J* = 4.2 Hz, 1H), 6.85 (d, *J* = 3.1 Hz, 1H), 3.06 (t, *J* = 7.5 Hz, 2H), 2.83 (t, *J* = 6.0 Hz, 4H), 2.78 (t, *J* = 7.5 Hz, 2H), 2.49 (t, *J* = 6.0 Hz, 2H); ^{13}C NMR (151 MHz, $CDCl_3$): δ 209.13, 142.37, 126.59, 124.73, 123.70, 58.62, 52.96, 41.25, 28.24.

4-(Phenylamino)-1-(2-(thiophen-2-yl)ethyl)piperidine-4-carbonitrile (4)

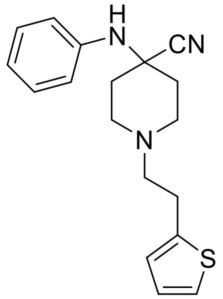

To a solution of **3** (4.0 g, 19.1 mmol) in AcOH (32.0 mL) was added aniline (1.9 mL, 21.1 mmol) at 0 ºC. Then TMSCN (2.7 mL, 21.2 mmol) dissolved in 6.5 mL of water was added dropwise at 0 ºC. The reaction mixture was stirred at 0 ºC for 1 h. Then it was allowed to slowly warm to room temperature and continued stirring for 3 h. After completion of the reaction, water (50 mL) was added and the pH adjusted to 10 by adding 10% aq. NH_4OH. The two layers were separated and the aqueous layer was extracted by dichloromethane. The combined organic layers were washed with brine solution and dried over anhydrous sodium sulfate, filtered and concentrated. The crude compound was purified by combiflash chromatography (0−15% ethyl acetate in CH_2Cl_2) to yield **4** (3.2 g, 53.8% yield) as an off-white solid. mp.100-102 ºC. ^{1}H NMR (600 MHz, $CDCl_3$): δ 7.27 – 7.25 (m, 2H), 7.14 (d, *J* = 5.0 Hz, 1H), 6.94 – 6.92 (m, 4H), 6.83 (d, *J* = 3.5 Hz, 1H), 3.66 (s, 1H), 3.02 (t, *J* = 7.5 Hz, 2H), 2.94 – 2.88 (m, 2H), 2.72 (t, *J* = 7.5 Hz, 2H), 2.54 (t, *J* = 11.0 Hz, 2H), 2.38 (d, *J* = 13.0 Hz, 2H), 1.96 (t, *J* = 10.5 Hz, 2H); ^{13}C NMR (151 MHz, $CDCl_3$): δ 143.21, 142.41, 129.29, 126.54, 124.66, 123.66, 120.95, 117.76, 59.15, 49.19, 36.07, 27.79.

4-(Phenylamino)-1-(2-(thiophen-2-yl)ethyl)piperidine-4-carboxamide (5)

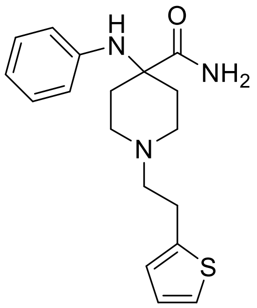

To a solution of **4** (3.0 g, 9.6 mmol) in DMSO (18.0 mL) was added K_2CO_3 (264 mg, 1.9 mmol). Then H_2O_2 (3.0 mL, 24.1 mmol) was added slowly dropwise at room temperature. The reaction mixture was stirred at room temperature for 16 h. Next, the mixture was filtered and the solid washed multiple times with diethyl ether and then dried under high vacuum. The crude solid **5** was used to next reaction without further purification. White solid (3.1 g, 97.8% yield); mp.173-175 ^oC. ^1H NMR (600 MHz, $CDCl_3$): δ 7.21 - 7.18 (m, 2H), 7.13 - 7.12 (dd, J = 1.2 Hz, 5.2 Hz, 1H), 6.92 - 6.89 (m, 2H), 6.83 - 6.80 (m, 2H), 6.66 – 6.64 (m, 2H), 5.30 (brs, 1H), 4.03 (s, 1H), 2.99 (t, J = 7.6 Hz, 2H), 2.85 - 2.82 (dt, J = 3.1 Hz, J = 12.2 Hz, 2H), 2.64 (t, J = 7.6 Hz, 2H), 2.39 - 2.34 (td, J = 4.1 Hz, 13.7 Hz, 2H), 2.20 - 2.15 (td, J = 1.9 Hz, 11.8 Hz, 1.97 (d, J = 12.7 Hz, 2H).

Methyl 4-(phenylamino)-1-(2-(thiophen-2-yl)ethyl)piperidine-4-carboxylate (6)

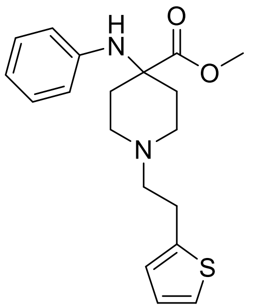

MeOH (12.0 mL) was placed in a sealed tube, which was then cooled to 0 ^oC. Triflic acid (1.7 mL, 19.7 mmol) was added slowly dropwise at 0 ^oC followed by addition of DMSO (0.3 mL). Next, amide **5** (500 mg, 1.5 mmol) was added in one portion and the reaction mixture was stirred at 0 ^oC for 5 minutes. Then the reaction mixture was refluxed in an oil bath at 80 °C for 2 days. After completion of the reaction, it was allowed to cool to room temperature and quenched with sat. a $NaHCO_3$ solution slowly by adding dropwise at 0 ^oC. The aqueous layer was washed with ethyl acetate twice (2 x 50 mL). The combined organic layers were washed with brine solution and dried over anhydrous sodium sulfate, filtered and concentrated. The crude product was purified by combiflash chromatography (0−1.5% methanol in CH_2Cl_2) to yield **6** (172 mg, 33% yield) as a pale-yellow viscous liquid. ^1H NMR (600 MHz, $CDCl_3$): δ 7.16 - 7.12 (m, 3H), 6.92 (t, J = 4.3 Hz, 1H), 6.82 (d, J = 3.1 Hz, 1H), 6.76 (t, J = 7.3 Hz, 1H), 6.57 (d, J = 8.1 Hz, 2H), 3.69 (s, 3H), 3.02 (t, J = 7.6 Hz, 2H), 2.70 - 2.65 (m, 4H), 2.50 (t, J = 10.3 Hz, 2H), 2.30 - 2.26 (m, 2H), 2.07 (d, J = 13.7 Hz, 2H); ^{13}C NMR (151 MHz, $CDCl_3$): δ 175.83, 144.84, 142.68, 129.13, 126.54, 124.59, 123.54, 118.65, 115.27, 59.71, 58.16, 52.35, 48.80, 32.93, 27.78.

(4-(Phenylamino)-1-(2-(thiophen-2-yl)ethyl)piperidin-4-yl)methanol (7)

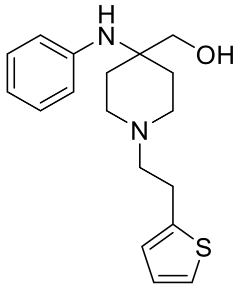

To a solution of **6** (200 mg, 0.6 mmol) in anhydrous THF (3.0 mL) was added $LiAlH_4$ (45 mg, 1.2 mmol) at 0 ºC. The reaction mixture was stirred at room temperature for 2 h. After completion of the reaction, it was quenched with H_2O/20% NaOH/H_2O (1:1:3). The reaction mixture was filtered with small Celite pad. The filtrate was washed with water and extracted with ethyl acetate. The combined organic layers were washed with brine solution and dried over anhydrous sodium sulfate, filtered and concentrated. The crude product was purified by combiflash chromatography (0 - 5% methanol in CH_2Cl_2) to yield **7** (173 mg, 94.5% yield) as an off-white solid. mp.103-105 ºC. ^{1}H NMR (600 MHz, $CDCl_3$): δ 7.20 (t, *J* = 7.9 Hz, 2H), 7.13 - 7.12 (m, 1H), 6.93 - 6.91 (m, 1H), 6.85 (t, *J* = 7.4 Hz, 1H), 6.82 - 6.79 (m, 3H), 3.64 (s, 2H), 3.02 (t, *J* = 7.8 Hz, 2H), 2.72 - 2.65 (m, 4H), 2.37 (t, *J* = 10.6 Hz, 2H), 1.96 (d, *J* = 13.9 Hz, 2H), 1.75 – 1.68 (m, 2H); ^{13}C NMR (151 MHz, $CDCl_3$): δ 145.01, 142.56, 129.21, 126.60, 124.62, 123.52, 120.02, 118.52, 59.97, 55.61, 49.05, 32,54, 27.74.

4-(Methoxymethyl)-N-phenyl-1-(2-(thiophen-2-yl)ethyl)piperidin-4-amine (8)

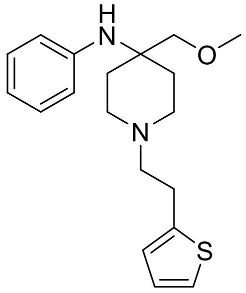

To a solution of NaH (23 mg, 0.96 mmol) in anhydrous THF (2.0 mL) in a sealed tube was added **7** (150 mg, 0.47 mmol) dissolved in 1.0 mL THF at 0 ºC. The reaction mixture was refluxed in an oil bath for 1 h at 70 ºC. Then the reaction mixture was allowed to cool to 0 ºC and MeI (60 µL, 0.95 mmol) was added in two portions. The reaction mixture was stirred at 0 ºC for 16 h. After completion of the reaction, it was quenched with H_2O (1.0 mL). The reaction mixture was filtered with a small Celite pad. The filtrate was washed with water and then extracted with ethyl acetate. The combined organic layers were washed with brine solution and dried over anhydrous sodium sulfate, filtered and concentrated. The crude product was purified by combiflash chromatography (0 - 2% methanol in CH_2Cl_2) to yield **8** (64 mg, 41% yield) as a brown viscous liquid. ^{1}H NMR (600 MHz, $CDCl_3$): δ 7.20 (t, *J* = 7.8 Hz, 2H), 7.15 (d, *J* = 5.0 Hz, 1H), 6.93 (t, *J* = 4.5 Hz, 1H), 6.91 - 6.87 (m, 2H), 6.82 (d, *J* = 7.6 Hz, 2H), 3.30 (s, 3H), 3.28 (s, 2H), 3.23 (t, *J* = 8.0 Hz, 2H), 2.98 - 2.80 (m, 6H), 2.03 - 1.97 (m, 4H); ^{13}C NMR (151 MHz, $CDCl_3$): δ 145.38, 128.97, 126.85, 125.20, 123.89, 120.74, 120.68, 59.49, 59.02, 54.78, 48.79, 31.80, 26.69.

Methyl 5-((4-(methoxymethyl)-1-(2-(thiophen-2-yl)ethyl)piperidin-4-yl)(phenyl)amino)-5-oxopentanoate (9)

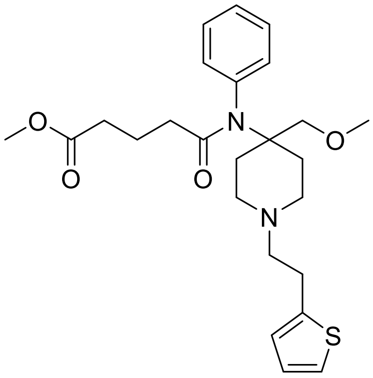

To a solution of **8** (55 mg, 0.17 mmol) in anhydrous dichloromethane (2.0 mL) in a sealed tube was added pyridine (27 µL, 0.34 mmol) and methyl 4- (chloroformyl)butyrate (41 mg, 0.25 mmol) at 0 ºC. The reaction mixture was refluxed in oil bath at 70 ºC for 16 h. Upon completion of reaction, it was quenched with a sat. $NaHCO_3$ solution. The mixture was extracted with CH_2Cl_2 (2 x 10 mL) and the combined organic layers were washed with brine, dried over anhydrous sodium sulfate, filtered and concentrated under reduced pressure provided a crude product, which was purified by flash column chromatography (0-1.5% methanol in dichloromethane) to obtain the **9** (48 mg, 72%) as a pale-yellow viscous liquid. ^{1}H NMR (600 MHz, $CDCl_3$): δ 7.44 - 7.39 (m, 3H), 7.35 (d, *J* = 7.5 Hz, 2H), 7.20 (d, *J* = 5.0 Hz, 1H), 6.91 (t, *J* = 4.3 Hz, 1H), 6.85 (d, *J* = 3.0 Hz, 1H), 4.05 (s, 2H), 3.58 (s, 3H), 3.43 (s, 3H), 3.04 (t, *J* = 8.0 Hz, 2H), 2.86 (d, *J* = 12.1 Hz, 2H), 2.78 (t, *J* = 8.0 Hz, 2H), 2.50 (t, *J* = 11.5 Hz, 2H), 2.26 (d, *J* = 13.5 Hz, 2H), 2.20 (t, *J* = 7.3 Hz, 2H), 1.89 (t, *J* = 7.3 Hz, 2H), 1.86 – 1.82 (m, 2H), 1.75 - 1.70 (qu, *J* = 7.3 Hz, 2H); ^{13}C NMR (151 MHz, $CDCl_3$): δ 175.32, 175.23, 141.84, 132.31, 130.13, 129.60, 127.89, 126.22, 124.79, 62.58, 60.50, 59.44, 51.97, 50.82, 37.08, 33.76, 33.09, 27.57, 21.77.

5-((4-(Methoxymethyl)-1-(2-(thiophen-2-yl)ethyl)piperidin-4-yl)(phenyl)amino)-5-oxopentanoic acid (10)

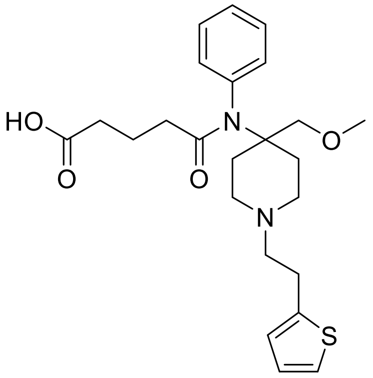

To a solution of ester **9** (40 mg, 0.09 mmol) in methanol 1.0 mL was added 0.3 mL of 1M LiOH•H_2O solution. The reaction mixture was stirred for 4 h at room temperature. Upon completion of reaction, the solution was acidified with 2N HCl in diethyl ether to pH 5. Then it was condensed in vacuo to remove methanol. The crude product was purified by column chromatography (0-5% methanol in dichloromethane) to provide **10** (17 mg, 37% yield) as an off-white solid. mp. 68-70 ºC. ^{1}H NMR (600 MHz, $CDCl_3$): δ 7.44 - 7.35 (m, 5H), 7.24 (d, *J* = 5.2 Hz, 1H), 6.93 (t, *J* = 4.2 Hz, 1H), 6.90 (d, *J* = 3.1 Hz, 1H), 4.07 (s, 2H), 3.44 (s, 3H), 3.16 - 3.07 (m, 6H), 2.87 (t, *J* = 11.1 Hz, 2H), 2.35 (d, *J* = 14.3 Hz, 2H), 2.11 (t, *J* = 7.2 Hz, 2H), 1.98 - 1.92 (m, 4H), 1.75 (qu, *J* = 7.3 Hz, 2H); ^{13}C NMR (151 MHz, $CDCl_3$): δ 178.92, 175.77, 141.61,

140.71, 132.29, 130.33, 129.76, 128.08, 126.77, 125.28, 61.74, 59.47, 59.31, 50.45, 37.55, 35.46, 32.05, 26.48, 22.35. HRMS (ESI): m/z $[M + H]^+$ calcd for $C_{24}H_{32}N_2O_4S$: 445. 2156; found: 445. 2164.

Sufentanil-BSA Conjugate (11)

A mixture of sufentanil acid **10** (0.1 mg, 2.0 equiv, 3.0 x 10^{-4} mmol), EDC (0.06 mg, 2.0 equiv, 3 x 10^{-4} mmol) and NHS (0.03 mg, 2.0 equiv, 3 x 10^{-4} mmol) in 45 µL of DMSO was prepared and stirred at room temperature for 16 h. Then the resulting reaction mixture was added drop wise to the solution of BSA (0.3 mg, 1 equiv, 1.35 x 10^{-4} mmol, dissolved in 300 µL water) and then stirred for 3 h. Next, a dialysis column (Thermo Scientific) was prepared by adding approximately 14.5 mL of sterile 1X PBS into the bottom reservoir. The top reservoir was then rinsed with 500 µL of 1X PBS and placed into the bottom and transferred the reaction mixture. Column was then sealed and placed onto an orbital shaker (100 rpm) for 2 hours. After agitation, the mixture was removed and placed into Eppendorf tubes and volume recorded. The mixture was then loaded into a 5 mL syringe and sterilize filtered through a 0.2 µm HT Tuffryn membrane (Acrodisk syringe filter, Pall Corporation, Ann Arbor, MI).

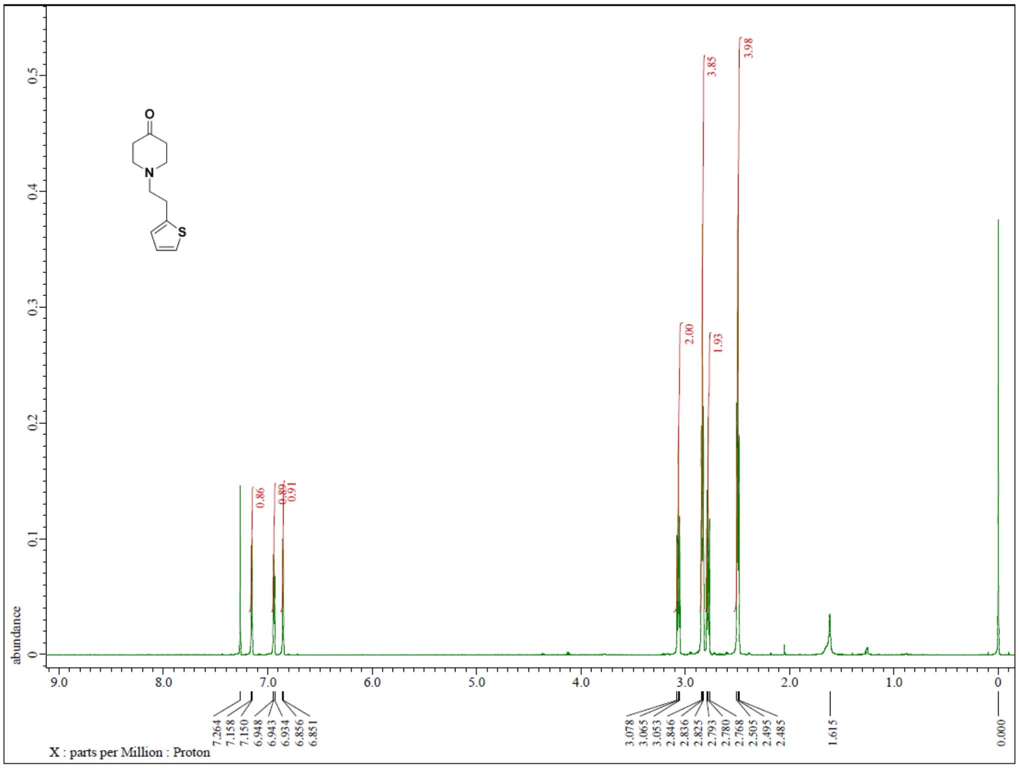

^1H NMR spectrum of **3** ($CDCl_3$, 600 MHz)

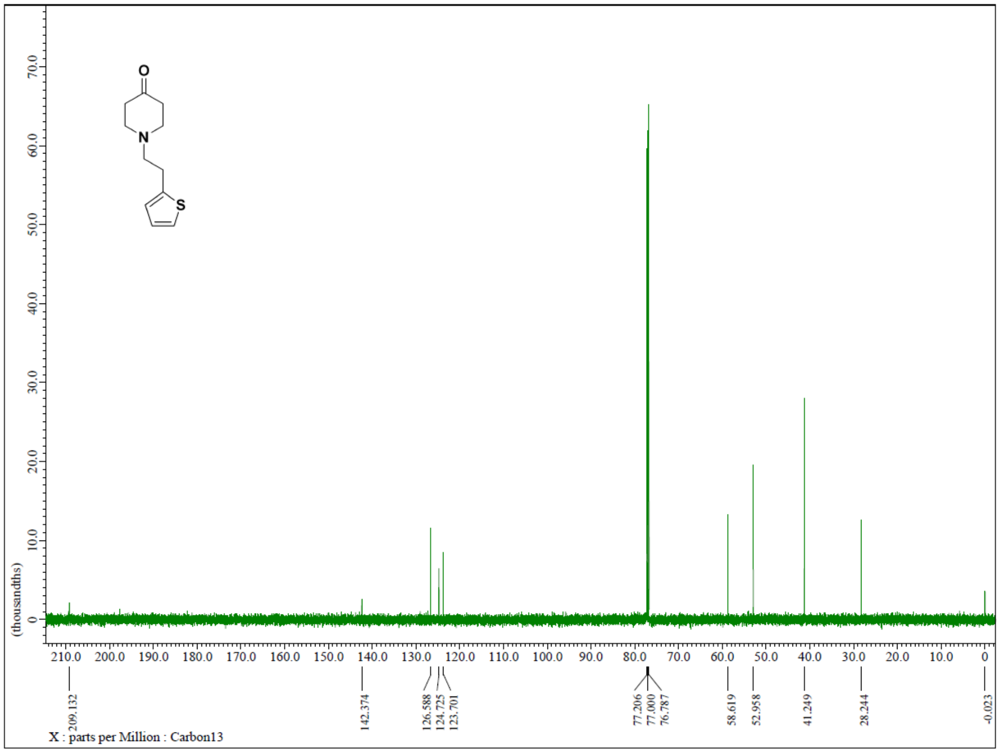

^{13}C NMR spectrum of **3** ($CDCl_3$, 151 MHz)

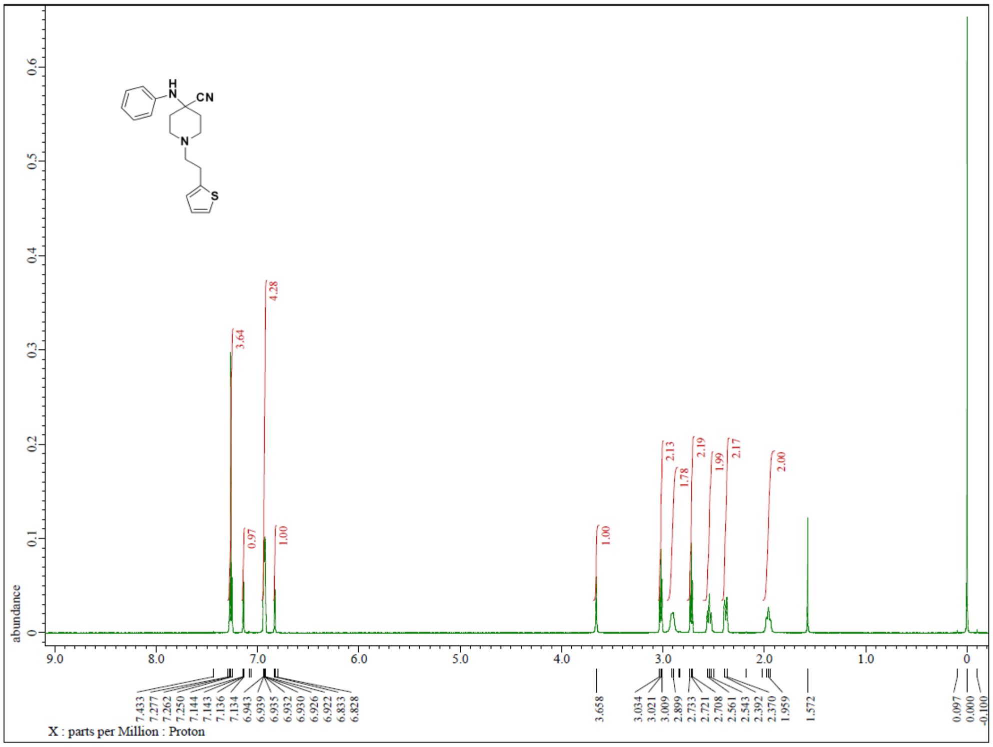

^{1}H NMR spectrum of **4** ($CDCl_3$, 600 MHz)

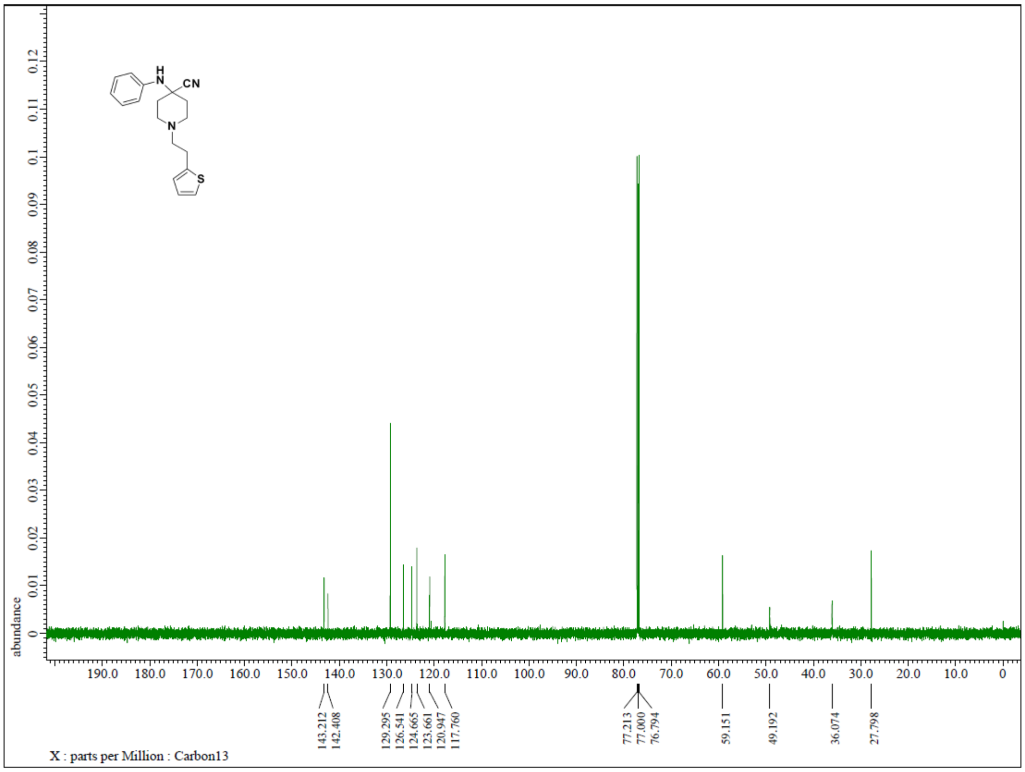

^{13}C NMR spectrum of **4** ($CDCl_3$, 151 MHz)

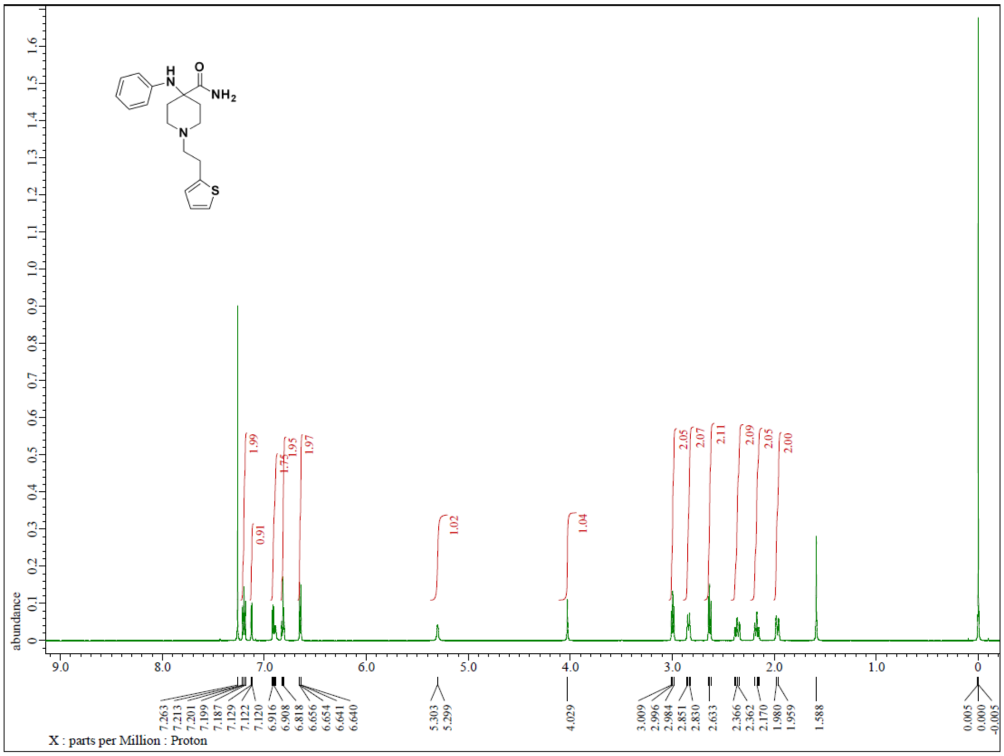

^{1}H NMR spectrum of **5** ($CDCl_3$, 600 MHz)

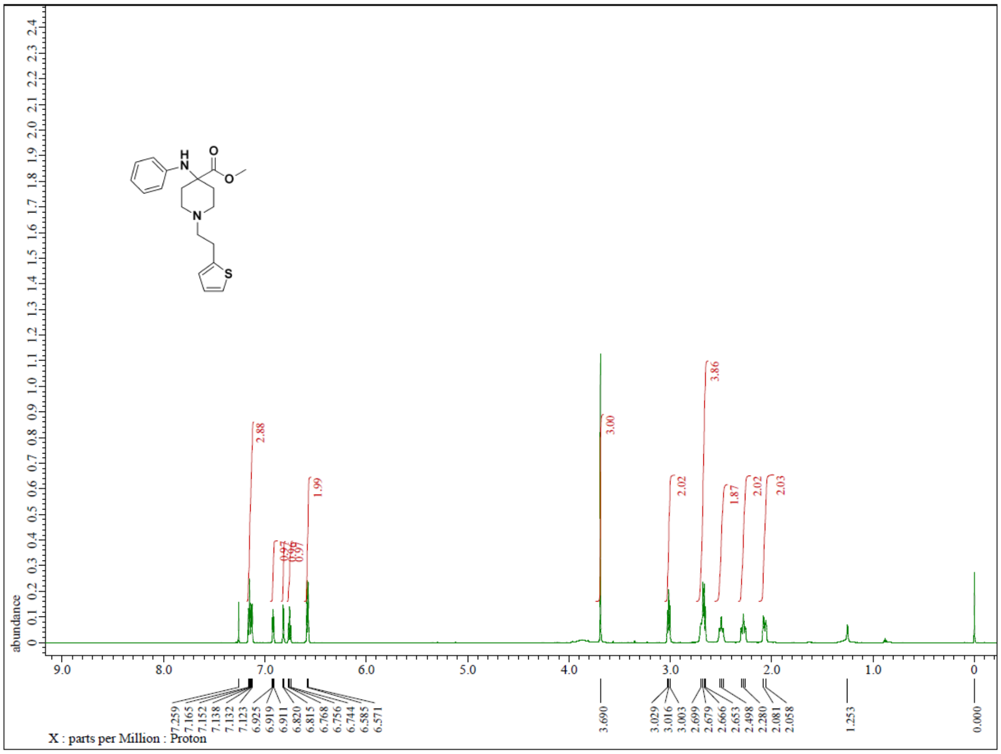

^{1}H NMR spectrum of **6** ($CDCl_3$, 600 MHz)

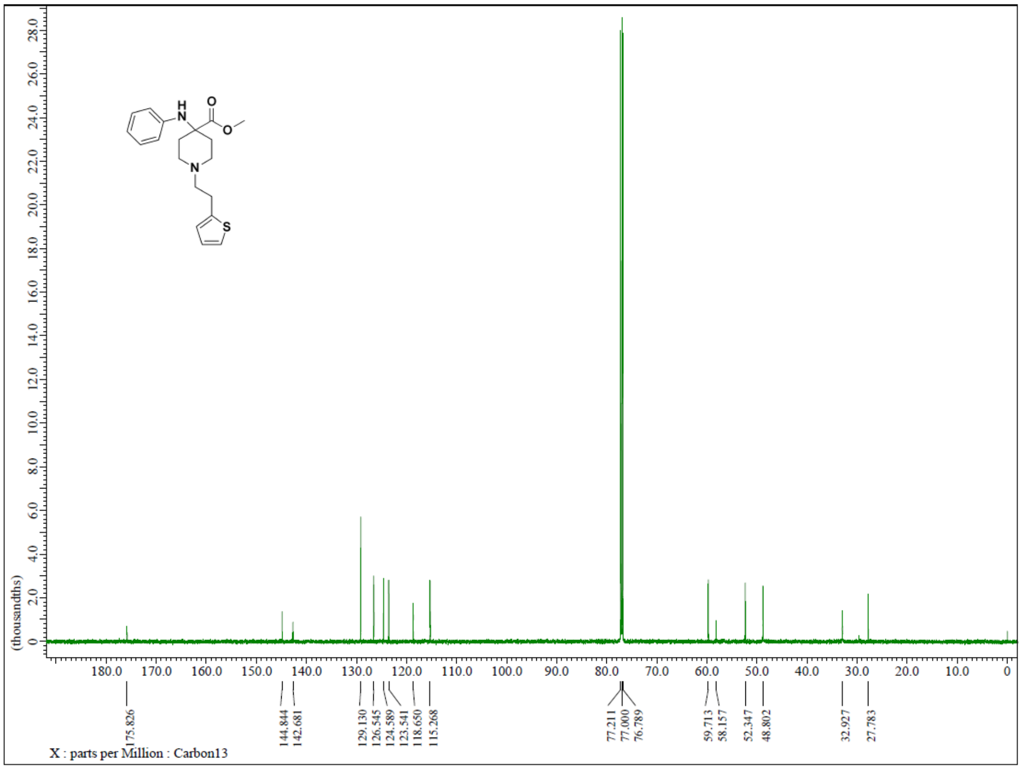

^{13}C NMR spectrum of **6** ($CDCl_3$, 151 MHz)

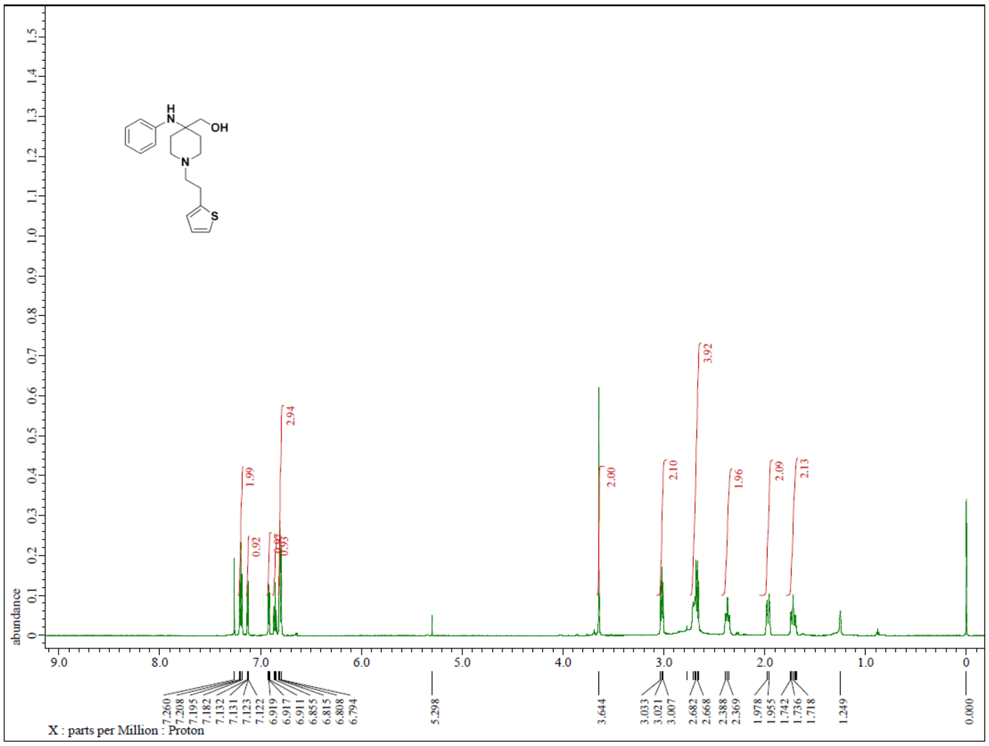

^{1}H NMR spectrum of **7** ($CDCl_3$, 600 MHz)

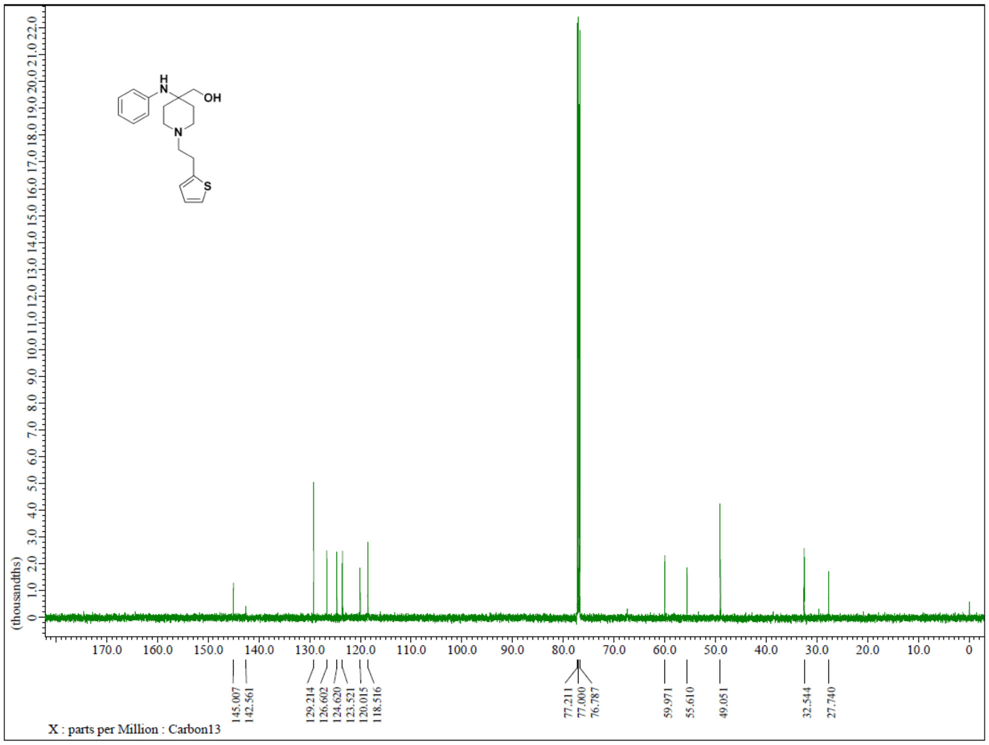

^{13}C NMR spectrum of **7** ($CDCl_3$, 151 MHz)

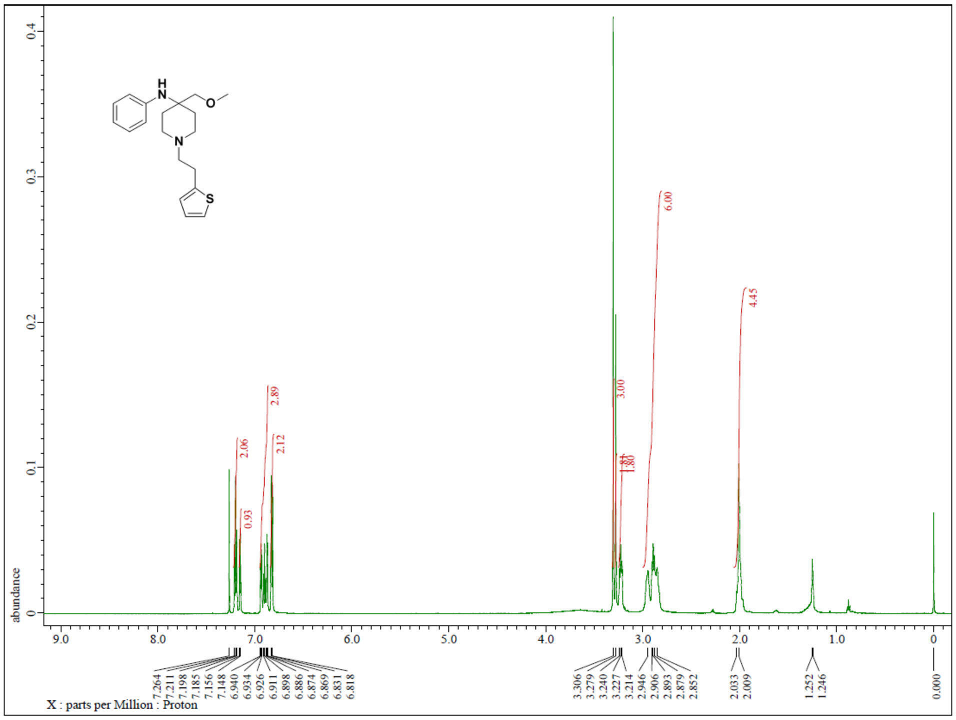

^{1}H NMR spectrum of **8** ($CDCl_3$, 600 MHz)

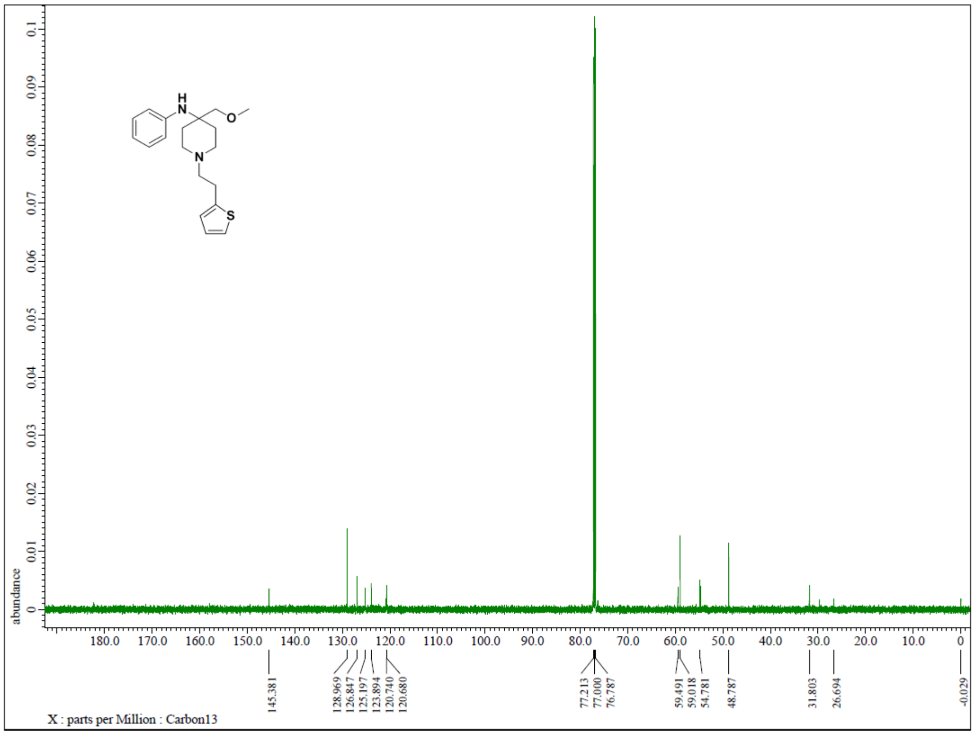

^{13}C NMR spectrum of **8** ($CDCl_3$, 151 MHz)

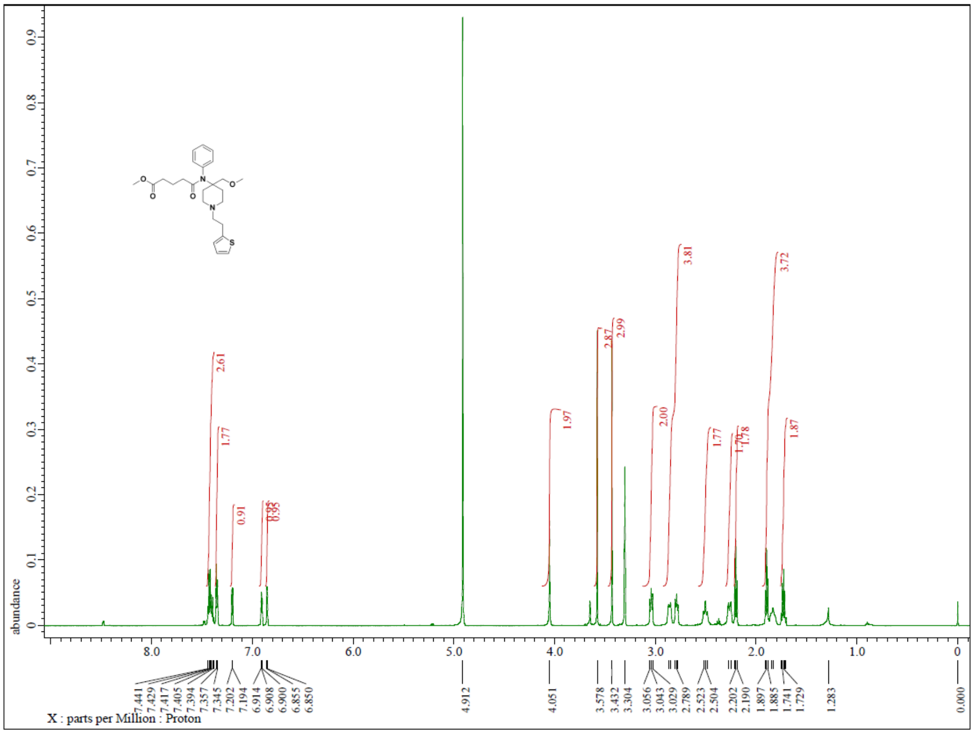

^{1}H NMR spectrum of **9** (MeOH-d_4, 600 MHz)

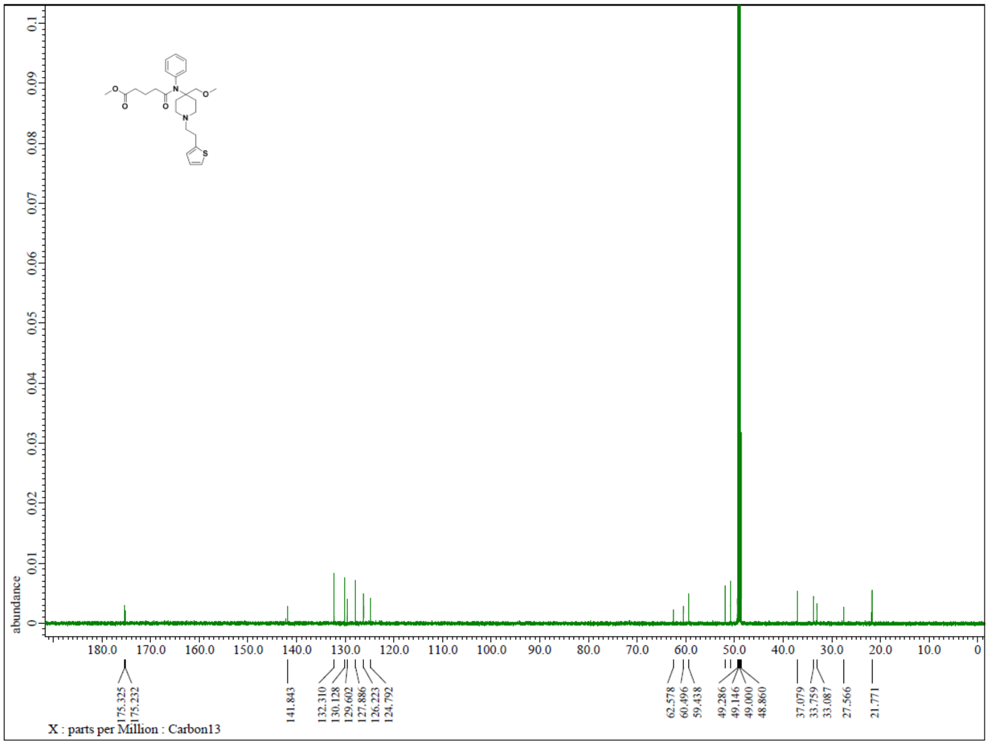

^{13}C NMR spectrum of **9** (MeOH-d_4, 151 MHz)

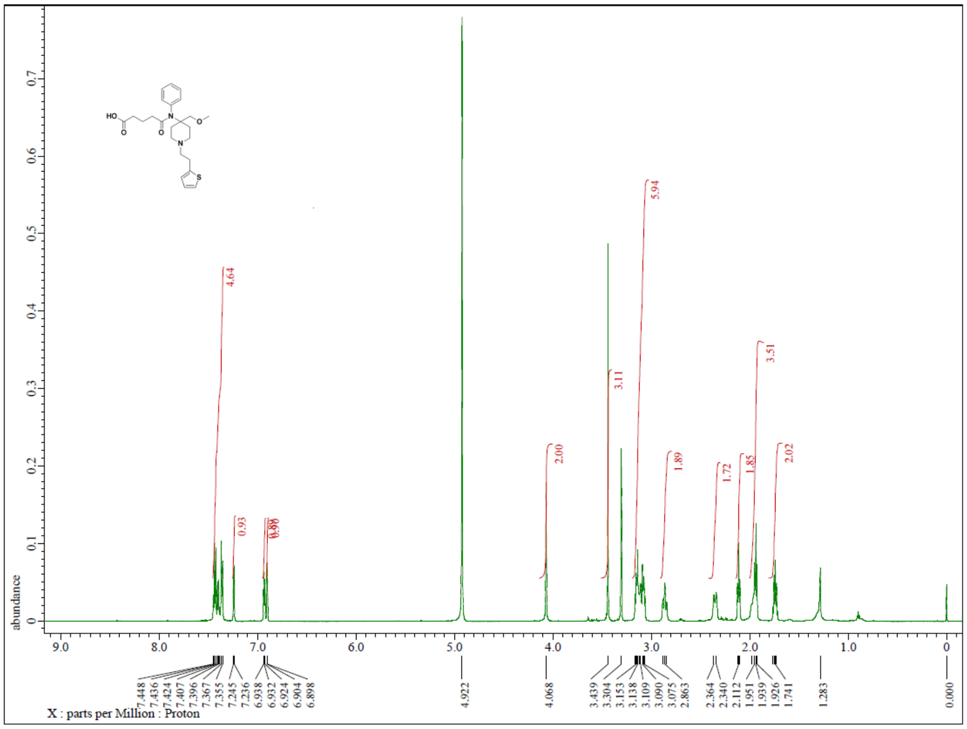

^{1}H NMR spectrum of **10** (MeOH-d_4, 600 MHz)

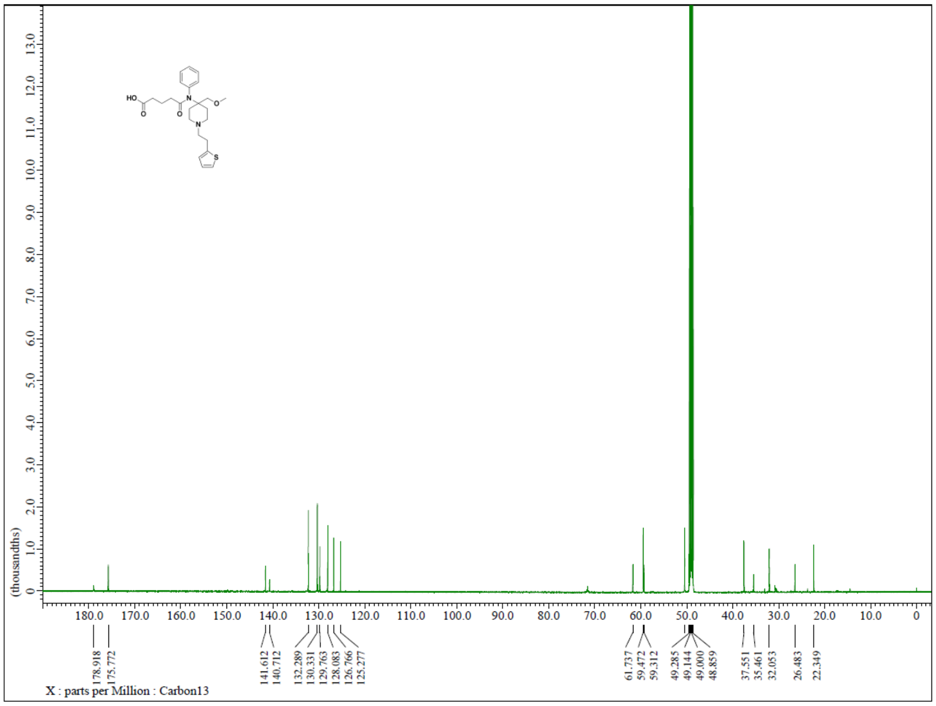

^{13}C NMR spectrum of **10** (MeOH-d_4, 151 MHz)
